# Peripheral Doses Beyond Electron Applicators in Conventional C-Arm Linear Accelerators: A Systematic Literature Review

**DOI:** 10.1177/15330338241239144

**Published:** 2024-03-22

**Authors:** Kapil Dev Maharaj, Joshua Dass, Mounir Ibrahim, Talat Mahmood, Pejman Rowshanfarzad

**Affiliations:** 1 School of Physics, Mathematics and Computing, 2720 The University of Western Australia, Crawley, Western Australia, Australia; 2 Department of Radiation Oncology, 5728 Sir Charles Gairdner Hospital, Nedlands, Western Australia, Australia; 3 Centre for Advanced Technologies in Cancer Research (CATCR), Perth, Western Australia, Australia

**Keywords:** peripheral dose, electron, therapy, applicator, linac, out-of-field

## Abstract

**Background:** This review investigates peripheral dose levels in electron beam treatments, comparing different manufacturers including Varian, Elekta, and Siemens. Accurate measurement of peripheral dose is vital for patient safety and precise radiation delivery in radiation therapy. **Methods:** This review followed PRISMA standards, conducting a comprehensive literature search from 1978 to July 2023. Emphasis was on identifying studies analyzing peripheral doses related to various electron beam energies, beam angle, field sizes, cutouts, and applicator combinations. Three major databases including PubMed, Web of Science, and Scopus were searched. **Results:** A total of 7 articles were included in this review. Strategies such as bolus materials, personalized cutouts, and optimal treatment procedures have all been developed to reduce peripheral radiation exposure and enhance patient safety. Ongoing research in this field is focused on further minimizing the risks associated with out-of-field radiation by improving dose delivery systems. **Conclusion:** The literature emphasizes importance of precision in electron beam radiation therapy, highlighting the critical need for managing peripheral doses and optimizing hardware to ensure patient safety. It advocates for the use of advanced tools and protocols to maintain a balance between effective treatment while protecting healthy tissues. Continuous research, careful treatment planning, and effective management of peripheral doses are essential.

## Introduction

Radiation therapy is a treatment option for primary, additional, or palliative treatment of skin cancer.^1^ Research indicates a control rate of 87% to 100% for skin tumors 2 to 5 years following radiation therapy^1^ which is the preferred approach for large, surgically complex, or inoperable tumors, and a valuable alternative for elderly patients.^1^ It can also be used postsurgery to eliminate the residual lesion and prevent recurrence. Approximately 50% of patients with cancer undergo radiation therapy, yet in low-income countries, late diagnoses and limited resources reduce this to 25% to 40%.^2,3^ Radiation therapy is an essential component of cancer treatment for all types of tumors, high-energy linacs made by Varian (Varian Medical Systems) and Elekta (Elekta AB) are most common linear accelerators used for electron and photon therapy at multiple energy levels. It should be noted that Siemens stopped manufacturing its Artiste, Oncor, and Primus linear accelerators since January 2012.

Radiotherapy options for skin cancer treatment include brachytherapy, electronic brachytherapy (intra-operative radiotherapy), kV X-ray therapy, and electron beam therapy.^1^ The choice of treatment option for each patient depends on the extent of the disease, its location, availability of equipment, and the radiotherapy team practices.^4^

Since the early 1950s, high-energy electron treatments have played a crucial role in radiation therapy as they have high linear energy transfer (LET) and cause more DNA chemical changes than photons, which can lead to damage in superficial cancerous cells,^5^ specifically those up to 5 cm deep, so, this method is notable for its marked reduction in dosage beyond the tumor's boundaries.^6^ In terms of biological effectiveness, radiation's biological efficacy, influenced by factors like LET, dose, and cell radiosensitivity, varies; high LET radiation (*ie,* electron) deposits more energy than its low LET (*ie,* photons) counterpart.^5,7^ The aim in radiation therapy is to maximize tumor control probability (TCP) while minimizing normal tissue complication probability (NTCP). It means effective tumor control while reduces side effects and harm to healthy tissues. Electron beam irradiation is a safe and highly effective treatment with a cure rate over 95%, making it a valuable option for skin cancer and an excellent treatment modality for epithelial skin cancer, especially in cosmetically sensitive areas, where surgery is less favorable. Additionally, it proves beneficial for postoperative treatment in case of residual or recurrent disease.^8^ The electron clinical markup remains the main approach to treating small skin cancers especially in centers that do not have a kilovoltage superficial unit or even more rarely a brachytherapy suite. The VMAT approach for treating a small 1 cm skin cancer such as a basal cell carcinoma (BCC) (which makes up more that 85% of all skin cancers) would be considered inappropriate use of resources and even acceptable dosimetry whereas the electron beam is an integral component of a linac and is readily available and deliverable.^8^ In electron beam therapy, an applicator constructed from an alloy material often utilized alongside Cerrobend cutouts to mold the electron beam, conforming it to the shape of the targeted area (*ie,* tumor).^9^ The cones are usually positioned 5 cm from the patient's skin surface when delivering treatment at a source-to-surface distance (SSD) of 100 cm.^10^ Cones provide collimation and ensure relatively sharp field edges and a flat radiation beam.^11^ However, specific irradiation techniques for example total skin electron irradiation^12^ stand as exceptions to this approach. The purpose of utilizing cones or applicators is to restrict the electron beam, reducing its lateral dispersion from the accelerator head to the patient's skin.^9^ In the process of electron beam therapy, as the electron beam passes through the scattering foil, the electrons scatter sufficiently when interacting with various components within the accelerator head, applicator, cutouts (inserts), and the air between the exit window and the patient. This scattering results in the emission of X-rays and production of low-energy electrons which causes the formation of clinically unacceptable penumbra. This leads to additional dose outside the treatment area, which is known as peripheral doses, as well as increased the absorbed dose.^12^ Furthermore, the electron component contributes to peripheral dose through 3 primary mechanisms: (1) scattering out of the applicator, (2) penetration of collimating structures within the applicator, and (3) direct exit into the surrounding air without interacting with applicator components.^13^ Notably, the latter 2 mechanisms—penetration and direct escape—are more common with higher-energy beams, while scattering is characteristic of lower-energy beams. Furthermore, as the energy surpasses 12 MeV, neutron contamination becomes prevalent.^14^ The neutron fluence escalates with the electron beam energy.^14^ These neutrons interact with nuclei in the LINAC head, bunker walls, and patient, triggering nuclear reactions. Resultant neutron capture can lead to prompt gamma-ray and nuclei activation, some decaying by β^−^ emission.^15-17^ Given neutrons’ high radiobiological effectiveness, their presence around LINACs in radiotherapy can contribute to nontrivial patient doses, potentially correlating with cancer relapse and new tumor initiation.^14^ Furthermore, such contamination poses an occupational hazard for facility staff, underscoring the importance of assessing risks associated with neutron exposure in these environments.^14^ However, studies by various groups, including Nath *et al*,^18^ Lin *et al*,^19^ Biltekin *et al*,^20^ and Cardenas *et al*,^21^ have corroborated that neutron contamination is less pronounced in electron beams compared to photon beams since the cross-section of electronuclear (e,n) reactions is smaller, by about 2 orders of magnitude, compared to photo-nuclear (γ,n) reactions.^22^ This difference is attributed to the lower bremsstrahlung efficiency, which means that a higher number of electrons is required to produce a certain number of photons.^22^ Additionally, photon beams penetrate tissues more deeply, requiring higher electron gun currents for electron irradiation. Consequently, by even only considering the differences in neutron generation cross-sections, the electron mode is expected to produce at least 2 orders of magnitude fewer neutrons compared to the photon mode.^22^

A comprehensive comparison of linear accelerators from Varian, Elekta, and Siemen vendors has been conducted.^23^ Each manufacturers create applicator designs and materials based on their unique linear accelerator gantry head configurations.^13,24,25^ Moreover, each vendor supplies a range of applicator sizes to accommodate different treatment fields sizes. In addition to this, for a more customized field shape, a lead or metal alloy cutout may be constructed and placed on the applicator as close to the patient as possible. Once an applicator is attached to the gantry head, the jaw settings are automatically locked through an integrated interlock system, ensuring consistent electron radiotherapy for that specific applicator and energy setting.

Recent advancements in external radiotherapy enable precise dose administration to target areas, reducing harm to adjacent healthy tissues.^26^ As a result, there has been an increase in overall survival rates for various cancer types.^27^ Long-term cancer survivors face a higher risk of subsequent malignancies.^28^ The incidence of secondary cancers has significantly increased due to a growing population of cancer survivors and an aging demographics, rising from 9% of all cancer diagnoses in 1975–1979 to 19% in 2005–2009.^29^ There is well-documented evidence indicating that secondary cancer risks are linked to radiotherapy and a study by Berrington *et al*^30^ has estimated that about 10% of secondary cancers in adults can be attributed to radiotherapy. However, the relationship between radiotherapy dose and possibility of secondary cancer induction is not straightforward. Existing models are based on the atomic bomb survivor data and are mainly applicable to photon treatments with large uncertainties.^31^ The out-of-field dose is usually considered insignificant in electron treatments; however, radiation carcinogenesis has no threshold and could even occur at doses below 0.1 Sv (ICRP 103).^32^ Measuring the electron out-of-field dose may enable the evaluation of possible damage to surrounding tissues in clinical cases such as: electron patch for breast and internal mammary chain (IMC) treatments, neck node treatments at extended SSD, testicular boost for total body treatment. Dose to pacemakers could also be affected.^9^ Hence, understanding peripheral doses is pivotal, especially when critical organs or the patient's skin are proximal to the applicator's side, as this knowledge enhances patient safety and optimizes treatment efficacy.

The aim of this review is to provide a comprehensive understanding of peripheral doses outside the applicator in electron beam therapy. This overview is provided by analyzing research on linear accelerators supplied by Varian, Elekta, and Siemens. Furthermore, significant findings pertaining to peripheral dose patterns beyond the applicator will be the central focus of present work. This examination will encompass variations resulting from factors such as beam energy, applicator design, field size, gantry angles, and depth. Additionally, mitigation strategies will be identified and characterized, including the utilization of bolus material, custom cutouts, and treatment method optimization. These strategies aim to elevate patient safety in the context of electron beam therapy.

This overview will benefit radiation oncologists, medical physicists, radiation therapists, and researchers. The findings are expected to instructions for treatment planning enhancement, optimization of dose delivery techniques, and the elevation of patient safety.

A systematic search of electronic databases such as PubMed and Web of Science was undertaken to identify recent and relevant publications. Key search terms included “electron beam therapy”, “linear accelerators”, and “peripheral dose”. The inclusion criteria encompassed dose assessments, distribution data, and potential mitigation techniques for peripheral exposure.

## Methods

In this review, a comprehensive literature exploration was undertaken, with a specific focus on articles written in English spanning the period from 1978 to July 2023. The exploration was conducted in accordance with the PRISMA standards. The emphasis of this exploration was placed on the identification of studies that delved into the analysis of peripheral doses concerning various electron beam energies and applicator pairings. Furthermore, the reference lists of all eligible papers were meticulously screened to uncover studies that might have been omitted through the initial search terms. Studies were excluded based on the following criteria: (1) peripheral or out-of-field doses not being central to the research; (2) absence of electron beam utilization; or (3) lack of involvement of applicators.

**Figure 1.**
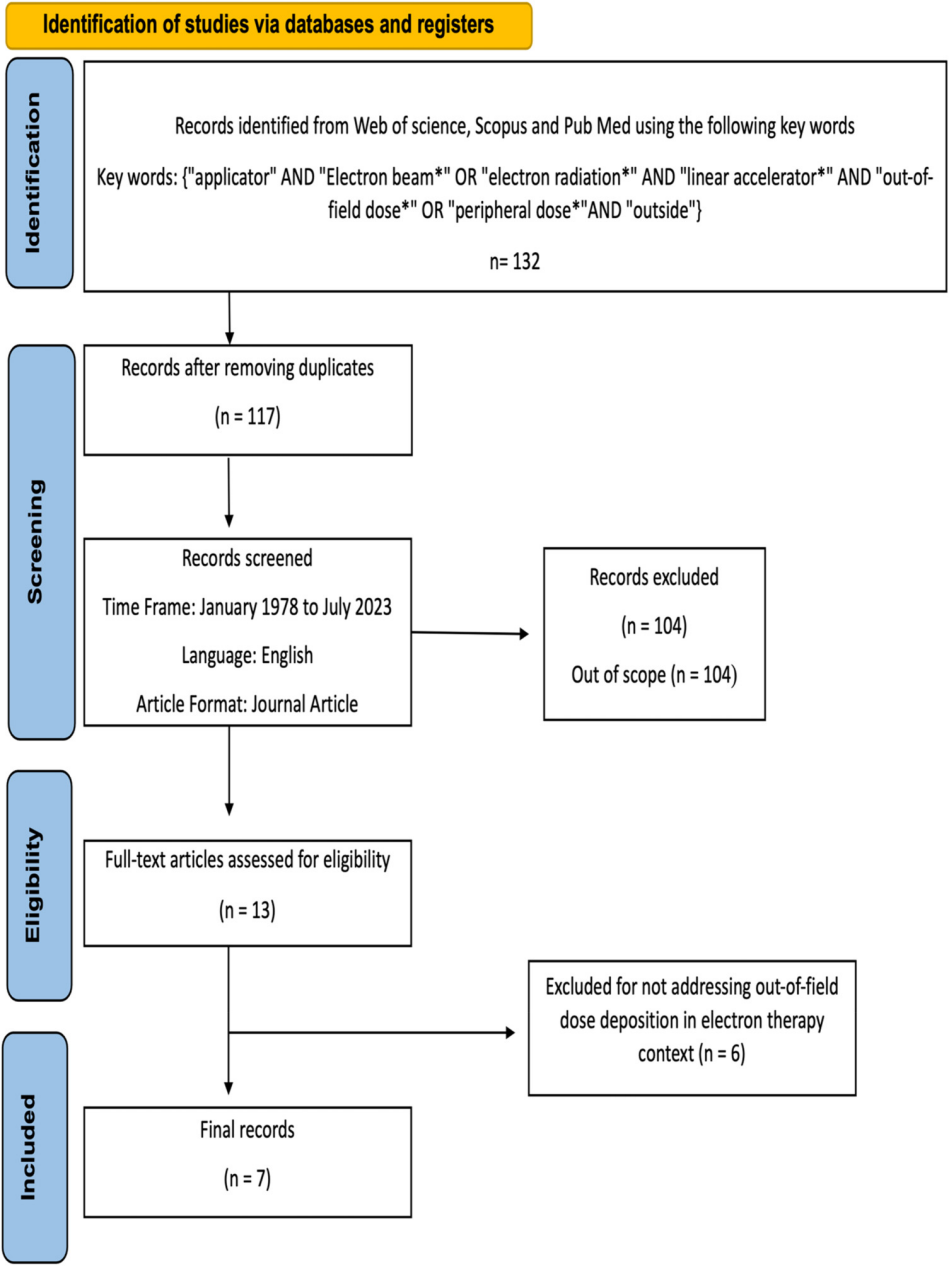
PRISMA flow diagram illustrating the systematic literature search and selection process.

Through a targeted search conducted across 3 prominent databases—PubMed, Web of Science, and Scopus—using predefined search parameters illustrated in Figure 1, a total of 132 relevant articles were identified. Initial screenings focused on titles and abstracts, resulting in the removal of unrelated or redundant studies. Subsequent to this initial selection, a comprehensive evaluation of the content within the remaining papers was carried out, ultimately resulting in a final selection of 7 articles that were deemed pertinent for inclusion in this review (as detailed in Table 1).

**Table 1.** Literature Review: Peripheral Doses Outside Applicator for Electron Therapy.

First author	Machine type	Electron beam energy (MeV)	Detector	Phantom	Applicator size (cm^2^)	Cutouts	Gantry angles (degrees)	Depth in phantom	Compared the data with TPS or MC	Recommendations
Van Der Laarse^33^	Philips SL-75 series	20	Polystyrene parallel plate and ionization chamber	Water phantom	10 × 10	Yes	0^0^	0.8 cm and d_max_	No	No
Richard A Keys^34^	Varian Clinac 20	6, 12, and 20	PTW ion chamber	Polystyrene solid water phantom	10 × 10 and 25 × 25	Yes	0^0^	1.3, 2.2, and 2	No	No
L J Van Battum^25^	Siemens Primus; Varian 230° C/D and Elekta Sli accelerator	10, 12, 18, and 21 (Siemens Primus); 6, 12,18, and 22 (Varian 230° C/D); and 6,10,12, and 14 (Elekta Sli)	Plane parallel ionization chamber	Water phantom	10 × 10 and 25 × 25	Not mentioned	0^0^	d_max_, R80, R50, and Rp	Beam MC	No
James CL Chow^13^	Varian 21 EX	4, 6, 9, 12, and 16	Kodak TL films	Solid water phantom	2 × 2; 4 × 4; 6 × 6 ; 8 × 8 and 10 × 10	Not mentioned	0^0^ to 5^0^; 10^0^ and 15^0^	0, 0.2, 0.5, and 1	No	One can reduce this dose by wrapping the base of the applicator with lead foil or applying extra shielding to the patient when needed. Professionals using low-energy electron beams in radiotherapy should remain informed about its behavior and properties beyond the applicator
Basak Iktueren^35^	Siemen Oncor	6, 9, and 15	Parallel plate ionization chamber	Water-equivalent water phantom	5 × 5, 10 × 10; 15 × 15 ; 20 × 20 and 25 × 25	Yes	0^0^; 10^0^ and 20^0^	Surface, 0.2, 0.5, and 1 and d_max_	TPS	TPS is not sufficient to measure the peripheral dose outside the applicators, and this dose can only be determined by direct measurement
Mohamad M.Alabdoaburas^36^	Siemen Oncor; Simen Primus; Varian 230° C/D	6, 9, 12, and 18	Thermoluminescent and powder dosimeters (TLD-700)	Water phantom	6 × 6 ;10 × 10; and 20 × 20	Not mentioned	0^0^	1 and 10	No	No
Abbas Haghparast^37^	Elekta Synergy	6, 10, and 18	EBT3 Film	Solid water phantom	6 × 6; 10 × 10; 14 × 14; and 20 × 20	Yes	0^0^, 10^0^, 20^0^	0, 0.5, and 1 and d_max_	No	It's advised to employ MC analyses to delve deeper into the off-target dose patterns of Elekta Synergy electron beams

Abbreviations: MC, Monte Carlo; TPS, treatment planning systems.

## Results

Number of papers has been published on radiation (leakage) outside the applicator.^7-9,15,16,21-32,34,38-41^ Measurements conducted through the utilization of ionization chambers,^9,21,24,25,33-35,38-43^ films,^13,34,37-40,44^ and thermoluminescent dosimeters^36,45,46^ to evaluate out-of-field radiation collimated by electron applicators. Van der Larese examines the scatter contribution of individual walls of MEL SL75-20 linear accelerator's applicator and proposed that the shape of the beam profile is determined by the 2 walls defining the dimension.^33^ Andrew *et al*,^45^ Richard A Keys *et al*,^34^ Edward et al,^39^ and Perec and Kubo et al^44^ investigated the radiation leakage at the surface of the applicator of Varian Clinac-18, Clinac-20, Clinac-2500 and Clinac-1800, respectively. Perec and Kubo, along with their collaborators, mitigated the leakage by adding a 1 mm thick layer of lead onto the applicator's surface. Edward *et al* found that in Clinac-18 and Clinac-20 Varian machine photon jaws were too wide allowing electron to escape through unshielded region of the electron applicator. Ebert *et al* investigated the transmitted and scattered radiation outside the radiation field of the Siemens KD2 linac using a Monte Carlo (MC) simulation method.^47^

Battum *et al* studied the scattered radiation from Varian 2300 C/D, Siemens Primus, and Elekta SLi electron applicators.^25^ In this study, it was discovered that scattered radiation at the surface of a water phantoms can be as high as 12%, which inversely depends on depth. While, with Varian applicators, it showed a dependency on beam energy. Moreover, Battum *et al* observed that applicators with a larger field size of 25 × 25 cm^2^ exhibit a reduced contribution of scattered radiation at the central axis (CAX) compared to those with a smaller field size of 10 × 10 cm^2^.

Chow and Grigorov^13^ reported the highest peak value of peripheral dose for Varian 21 EX, approximately 1% of central-axis d_max_, at a distance of about 12 cm from the CAX on a film placed on the surface of a solid water phantom (SWP). This measurement was obtained using a 10 × 10 cm² applicator and a cutout for the 4 MeV electron beam along both the cross-plane and in-plane axes. Furthermore, this study revealed that the peripheral peak dose at a depth of 1 cm in the SWP increased proportionally with the angle of obliquity. Specifically, the local peak dose escalated by approximately 3% for each degree of increase in angle, causing a displacement of about 7 mm toward the CAX as the angle increased from 0 to 15°. The peak surface dose was positioned approximately 12 cm away from the CAX. At a depth of 1 cm and at the location of the peak dose spot, the peripheral dose was measured to be approximately 1% of the prescribed dose when using the 10 × 10 cm² applicator and cutout.

Peripheral dose value for Oncor Linear accelerator was measured by Iktueren et al.^35^ Author observed 1.4% dose peak (profiles were normalized to the CAX of the treatment field), located 6 cm away from the field edge, with a 10 × 10 cm², where the CAX received 100% dose. This phenomenon occurred at a gantry angle of 0° for both 6 and 9 MeV electron beams. In the case of the 15 MeV electron beam, a 2.3% dose peak was observed under similar conditions. Further investigations revealed that the peak dose reduced as the depth increased, reaching values between 2.5% to 4% of central axis d_max_ depending on the size of the treatment field. At gantry angles of 10° and 20°, 6 and 9 MeV electron beams exhibited small peaks, with maximum doses observed at depths of 0.2 and 1 cm. In contrast, the 15 MeV electron beam did not exhibit peaks at depths of 0.2 and 1 cm when the gantry angles were set at 10° and 20°. In addition to this, comparisons were made between the measured peripheral doses outside the applicators and the data generated by a treatment planning system (TPS) using the pencil beam algorithm. These comparisons indicated that dose calculations could be reliably performed up to a distance of 3 cm outside the treatment field.

Alabdoaburas et al^36^ investigated the out-of-field doses in water phantom for Siemen Oncor, Simen Primus, and Varian 2300 C/D. Notably, when Siemens applicators were used, a distinct peak dose was identified at approximately 12 to 15 cm beyond the field edge, specifically at a depth of 1 cm. This phenomenon was consistent across various field sizes and electron beam energies. For the Siemens Primus linac equipped with a 10 × 10 cm² applicator, the peak dose reached 2.3%, 1%, 0.9%, and 1.3% of the maximum central axis dose (d_max_) for 6, 9, 12, and 18 MeV electron beams, respectively. As for the Siemens Oncor linac with the same 10 × 10 cm² applicator, the peak dose values were 0.8%, 1%, 1.4%, and 1.6% of d_max_ for 6, 9, 12, and 14 MeV electron beams, respectively, and these values increased with larger applicator sizes. On the other hand, when considering the Varian 2300C/D linac, the doses measured at 12.5 cm beyond the field edge were 0.3%, 0.6%, 0.5%, and 1.1% of d_max_ for 6, 9, 12, and 18 MeV electron beams, respectively, and these values also showed an increase with larger applicator sizes. Notably, no distinct peak dose was observed for the Varian applicator across these energy levels. These findings provide valuable insights into the out-of-field dose characteristics associated with these linacs and applicators, which can contribute significantly to our understanding of radiation therapy delivery.

Haghparast et al^37^ measured the peripheral does for Elekta Synergy linac. For this study, the author normalized the peripheral dose profiles to a distance of 2 cm from the edge of each treatment field. Notably, the highest peak in the peripheral dose was observed when using an 18 MeV electron beam, located 3 cm from the outer edge of the applicator. This peak dose increased as the energy level of the electron beam increased. Specifically, when a 20 × 20 cm² applicator was employed, the peak dose for an 18 MeV electron beam reached 1.6% at the surface of the phantom and at a distance of 2 cm from the outer edge of the applicator. In comparison, for a 6 MeV electron beam within the same applicator size, the peak dose at the same distance was measured at 1.15%. It was observed that the peak dose diminished as the depth increased and increased with an increase in the size of the treatment field. Additionally, the peak dose shifted closer to the central beam axis with an increase in the gantry angle, similar result was also observed by Chow and Grigorov^13^ with Varian 21 EX linac.

## Discussion

Electron beam radiation therapy continues to serve as an effective method for treatment of skin cancer, prioritizing precise targeting and minimal impact on adjacent healthy tissues.

During the course of electron beam treatment, radiation leakage from the accelerator head and scattering originating from both the accelerator components and the patient contribute to the deposition of radiation dose in the tissues beyond the intended treatment area.^30^ For instance, accurate knowledge of out-of-field radiation doses in radiation therapy is essential for assessing the effect on healthy tissues. In patients with potentially adjacent organs at risk peripheral dose could lead to adverse effects such as cataracts, or secondary malignancies when using photon beam.^31^ To develop a risk model to estimate the effects of out-of-field dose on inducing secondary cancers, further epidemiologic studies and detailed out-of-field dose information are required, presenting a significant challenge^31^ especially as patients are not usually monitored for the out of field doses.

Although electron applicators primarily absorb electrons, their interactions with gantry components (*eg,* ion chambers), applicator, cutout, and the air gap between the patient and the applicator's end result in X-ray photons. These photons subsequently produce secondary electrons. Both these scattered electrons and photons can either deviate outside the treatment region or intensify the absorbed dose.^12^

In Battum *et al* found that Elekta SLi applicator yield lower scatter compared to Varian 2300/CD and Siemens Primus but have a pronounced effective angular discrepancy. In a previous study by Van Battum *et al,*^48^ it was observed that Elekta applicators displayed broader penumbras due to increased initial angular variance. While modifications in applicator design can reduce scattered radiation, they may also enhance this angular variance. Furthermore, in comparison to previous measurements conducted on a CGR Sagittaire accelerator equipped with a scanning beam and a trimmer system^25,49^ the scattered radiation from the applicators was notably higher for all 3 accelerators.

Meanwhile, Chow and Grigorov’s^13^ investigated the dependence of peripheral dose on the angle of obliquity, cutout and applicators sizes of Varian 21 EX linac. Their findings showed that peripheral doses increase with angular changes (0.81, 0.95, 1.09, and 1.27 cGy for 0, 5, 10, and 15 gantry angles, respectively), highlighting the need for extra care during treatments, especially when using low-energy electron beams. Furthermore, Chow *et al.* mentioned that electron beams are not likely to escape or penetrate the applicator. However, the major source of peripheral dose could be the electron scattering. To verify this, the authors recommended using MC simulation.^13^

Studies conducted by Iktueren *et al*,^35^ Alabdoaburas *et al,*^36^ and Haghparast *et al*^37^ sheds light on the factors affecting the peripheral doses, such as depth, field size, applicator size, gantry angle, and beam energy. A significant observation made by Iktueren *et al* was the limitation of TPS in accurately estimating peripheral doses, which introduces a clinical risk concerning the irradiation of sensitive organs like the eyes.

This concern is particularly significant for low-energy electron beams, such as 6 MeV, which are often used for treating surface lesions. Alabdoaburas *et al*^36^ also emphasized the necessity for TPS to account for these out-of-field doses, ensuring the safety of peripheral organs, particularly when a peak dose occurs.

The contribution of radiation scattered and transmitted by the applicator to the dose outside the radiation field has been investigated using MC simulation. For instance, Ebert *et al*^47^ analyzed the scattered and transmitted radiation outside the radiation field of the Siemens KD2 linear accelerator using the EGS4 MC method. They found that the major perturbation in the incident beam characteristics caused by an applicator is from scattered electrons, and the fluence and energy characteristics of scattered electrons were dependent on the primary beam energy and the applicator and aperture configuration. In another study, Tomohiro *et al*^12^ investigated the peripheral dose outside the radiation field of a Varian Clinac 2100CD applicator for a range of energies (4, 6, 9, 12, and 16 MeV) using a detailed MC simulation in EGSnrc at 5 mm depth in a water phantom for the 10 × 10 cm^2^ applicator. They found approximately 3% to 2% of dose was received from the field edge up to 2 cm, and about 1% of the dose was received beyond the 2 up to 12 cm from the field edge. These results were in agreement with an independent experimental study conducted by Chow *et al*, confirming that the peripheral dose outside the field is significantly affected by radiation scattered or transmitted from the applicator, and the effect increases with electron beam energy.

The breadth of existing research on electron beam radiation therapy underscores its important role in treating various cancers while emphasizing the complex considerations around peripheral doses, accessories selection, and energy variability. Literatures demonstrate the criticality of precise treatment planning to mitigate the risks associated with unintentional irradiation of tissues adjacent to the targeted malignancy. Key factors influencing peripheral doses include the type of linear accelerator, applicator size, beam energy, and even the depth at which the tumor is located. These considerations are pivotal for developing robust TPS that can accurately capture both in-field and out-of-field doses.

The mitigation of peripheral doses in electron beam radiotherapy has gained considerable scholarly attention.^9,13,21,46^ Chow and Grigorov^13^ have proposed the utilization of lead foil either to wrap the lower section of the applicator or to position additional shielding directly on the patient. They discussed that awareness of the dose properties outside the applicator is crucial, especially when employing low-energy electron beams in treatments.

Similarly, Yeboah *et al*^9^ investigated the modification of the Siemens Primus applicator's sidewalls by adding a 1 mm thick lead sheet. Their findings indicated considerable reductions in peak peripheral doses, amounting to 80% and 74% for incident electron beams of 9 and 18 MeV, respectively. The study underlines that their choice of a 1 mm lead thickness not only prioritizes weight considerations but also minimizes interference with clinical electron beams. Cardenas *et al*^21^ take a different approach, suggesting that applying a water-equivalent bolus with a thickness determined by E(MeV)/4 could significantly reduce out-of-field doses. Their study indicates that such a bolus reduces out-of-field doses to <0.5% up to a depth equal to the central-axis d_max_ for a given electron energy. Huifang *et al*^46^ explored the use of lead aprons (LAs) for shielding and noted that increasing the number of LA layers and the distance from the central beam axis significantly diminished out-of-field doses. In particular, for 4 MeV the use of LAs reduced the doses by up to approximately 98% at 30 cm from the central beam axis in comparison to scenarios without shielding.

## Conclusion

The literature supports the need for accuracy in electron beam radiation therapy. Although each electron applicator is specifically designed to provide a flat field for a specified depth at a specific SSD, it is important to verify the out-of-field doses for each applicator. Advanced tools, equipment, and protocols are essential for maintaining a delicate balance between effective treatment and patient safety. With the advancement of technology, continuous research, and careful treatment planning are essential. For accurate radiation therapy, it is important to conduct a comprehensive analysis on each linear accelerator due to differences in internal structure of the linac head and applicator geometries, for each linac model and manufacturer. Employing advanced computational methods, such as the GEANT4 MC simulation, facilitates a nuanced understanding of beam particle interactions. MC simulations can elucidate the complex scattering mechanisms inherent in radiation therapy, thereby enabling the development of strategies to limit the undesired scattered radiation. Effective management of peripheral doses is intricate, requiring precise tools and techniques to balance the treatment efficacy with potential risks. The overarching goal is to target the disease effectively while safeguarding the surrounding healthy tissues.

## Abbreviations

BCC: basal cell carcinoma
CAX: central axis
HDR: high dose rate
LET: linear energy transfer
MC: Monte Carlo
NTCP: normal tumor control probability
SWP: solid water phantom
TCP: tumor control probability
TPS: treatment planning system
VMAT: volumetric arc therapy.

## References

[1] Pashazadeh A Boese A Friebe M . Radiation therapy techniques in the treatment of skin cancer: an overview of the current status and outlook. J Dermatol Treat. 2019;30(8):831-839. doi:10.1080/09546634.2019.1573310 30703334

[2] Hanna T Shafiq J Delaney G Vinod S Thompson S Barton M . The population benefit of evidence-based radiotherapy: 5-year local control and overall survival benefits. Radiother Oncol. 2018;126(2):191-197. 29229506 10.1016/j.radonc.2017.11.004

[3] Rosenblatt E Zubizarreta E . *Radiotherapy in cancer care: facing the global challenge*. International Atomic Energy Agency Vienna; 2017.

[4] van Hezewijk M Creutzberg CL Putter H , et al. Efficacy of a hypofractionated schedule in electron beam radiotherapy for epithelial skin cancer: analysis of 434 cases. Radiother Oncol. 2010;95(2):245-249. 20334941 10.1016/j.radonc.2010.02.024

[5] Baskar R . Emerging role of radiation induced bystander effects: cell communications and carcinogenesis. Genome Integr. 2010;1(1):1-8. 20831828 10.1186/2041-9414-1-13 PMC2949714

[6] Khan FM Gibbons JP . Khan's the physics of radiation therapy. 5th Edition ed. Lippincott Williams & Wilkins; 2014.

[7] Hall EJ . Cancer caused by x-rays—a random event? Lancet Oncol. 2007;8(5):369-370. 17466892 10.1016/S1470-2045(07)70113-4

[8] Janiszewska M Raczkowski M Walczak J Składowski K Maciejczyk A . The physical and clinical aspects of radiation therapy in skin cancer and subcutaneous tissue neoplasm. Health. 2018;10(6):730-748.

[9] Yeboah C Karotki A Hunt D Holly R . Quantification and reduction of peripheral dose from leakage radiation on Siemens Primus accelerators in electron therapy mode. J Appl Clin Med Phys. 2010;11(3):154-172. 10.1120/jacmp.v11i3.3105 PMC5720440 20717080

[10] Podgoršak EB . Radiation physics for medical physicists. In: Becker KH Di Meglio J-M Hassani S , et al. eds. Biological and medical physics, biomedical engineering. Springer; 2006.

[11] Salanitro PR . Electron dosimetry and treatment. In: Brady LW Yaeger TE , eds. Encyclopedia of radiation oncology. Springer; 2013:198-206.

[12] Shimozato T Okudaira K Fuse H Tabushi K . Monte Carlo simulation and measurement of radiation leakage from applicators used in external electron radiotherapy. Phys Med. 2013;29(4):388-396. 22771332 10.1016/j.ejmp.2012.06.006

[13] Chow JC Grigorov GN . Peripheral dose outside applicators in electron beams. Phys Med Biol. 2006;51(12):N231. 10.1088/0031-9155/51/12/N01 16757855

[14] Soto-Bernal TG Baltazar-Raigosa A Medina-Castro D Vega-Carrillo HR . Neutron production during the interaction of monoenergetic electrons with a tungsten foil in the radiotherapeutic energy range. Nucl Instrum Methods Phys Res Sect A. 2017;868:27-38.

[15] Bieniasiewicz M Konefał A Wendykier J Orlef A . Measurements of thermal and resonance neutron fluence and induced radioactivity inside bunkers of medical linear accelerators in the center of oncology in Opole, Poland. 2016.

[16] Konefał A Orlef A Bieniasiewicz M . Measurements of neutron radiation and induced radioactivity for the new medical linear accelerator, the Varian TrueBeam. Radiat Meas. 2016;86:8-15.

[17] Vega-Carrillo HR de Leon-Martinez HA Rivera-Perez E Benites-Rengifo JL Gallego E Lorente A . Induced radioisotopes in a linac treatment hall. Appl Radiat Isot. 2015;102:103-108. 25989748 10.1016/j.apradiso.2015.05.004

[18] Nath R Meigooni AS King CR Smolen S d'Errico F . Superheated drop detector for determination of neutron dose equivalent to patients undergoing high-energy x-ray and electron radiotherapy. Med Phys. 1993;20(3):781-787. 8350837 10.1118/1.597145

[19] Lin J-P Chu T-C Lin S-Y Liu M-T . The measurement of photoneutrons in the vicinity of a Siemens Primus linear accelerator. Appl Radiat Isot. 2001;55(3):315-321. 11515653 10.1016/s0969-8043(01)00084-7

[20] Biltekin F Yeginer M Ozyigit G . Investigating in-field and out-of-field neutron contamination in high-energy medical linear accelerators based on the treatment factors of field size, depth, beam modifiers, and beam type. Phys Med. 2015;31(5):517-523. 25873196 10.1016/j.ejmp.2015.03.015

[21] Cardenas CE Nitsch PL Kudchadker RJ Howell RM Kry SF . Out-of-field doses and neutron dose equivalents for electron beams from modern Varian and Elekta linear accelerators. J Appl Clin Med Phys. 2016;17(4):442-455. 10.1120/jacmp.v17i4.6216 PMC5690067 27455499

[22] Expósito M Romero-Hermida M Terrón J , et al. Neutron contamination in medical linear accelerators operating at electron mode. Springer; 2013:1225-1228.

[23] Zhu TC Wang KK-H . Linear accelerators (LINAC). In: Brady LW Yaeger TE , eds. Encyclopedia of radiation Oncology. Springer; 2013:437-450.

[24] Kassaee A Altschuler M Ayyalsomayajula S Bloch P . Influence of cone design on the electron beam characteristics on clinical accelerators. Med Phys. 1994;21(11):1671-1676. 7891626 10.1118/1.597280

[25] Van Battum L Van der Zee W Huizenga H . Scattered radiation from applicators in clinical electron beams. Phys Med Biol. 2003;48(15):2493. 12953911 10.1088/0031-9155/48/15/316

[26] Chargari C Soria J-C Deutsch E . Controversies and challenges regarding the impact of radiation therapy on survival. Ann Oncol. 2013;24(1):38-46. 22898033 10.1093/annonc/mds217

[27] Arnold M Rutherford MJ Bardot A , et al. Progress in cancer survival, mortality, and incidence in seven high-income countries 1995–2014 (ICBP SURVMARK-2): a population-based study. Lancet Oncol. 2019;20(11):1493-1505. 31521509 10.1016/S1470-2045(19)30456-5 PMC6838671

[28] Dracham CB Shankar A Madan R . Radiation induced secondary malignancies: a review article. Radiat Oncol J. 2018;36(2):85. 29983028 10.3857/roj.2018.00290 PMC6074073

[29] Morton LM Onel K Curtis RE Hungate EA Armstrong GT . The rising incidence of second cancers: patterns of occurrence and identification of risk factors for children and adults. Am Soc Clin Oncol Educ Book. 2014;34(1):e57-e67. doi:10.14694/EdBook_AM.2014.34.e57 24857148

[30] De Gonzalez AB Curtis RE Kry SF , et al. Proportion of second cancers attributable to radiotherapy treatment in adults: a cohort study in the US SEER cancer registries. Lancet Oncol. 2011;12(4):353-360. 21454129 10.1016/S1470-2045(11)70061-4 PMC3086738

[31] Kry SF Titt U Pönisch F , et al. A Monte Carlo model for calculating out-of-field dose from a Varian beam. Med Phys. 2006;33(11):4405-4413. 17153419 10.1118/1.2360013

[32] Valentin J . International commission on radiological protection. The 2007 recommendations of the international commission on radiological protection. Ann ICRP, ICRP Publ. 2007;103:2-4. 10.1016/j.icrp.2007.10.003 18082557

[33] Van der Laarse R Bruinvis I Nooman MF . Wall-scattering effects in electron beam collimation. Acta Radiol Oncol Radiat Phys Biol. 1978;17(2):113-124. 99983 10.3109/02841867809127912

[34] Keys RA Purdy JA . Radiation leakage from linac electron applicator assembly. Int J Radiat Oncol Biol Phys. 1984;10(5):713-721. 6429095 10.1016/0360-3016(84)90302-x

[35] Iktueren B Bilge H Karacam S Atkovar G . The peripheral dose outside the applicator in electron beams of Oncor linear accelerator. Radiat Prot Dosim. 2012;150(2):192-197. 10.1093/rpd/ncr392 22025738

[36] Alabdoaburas MM Mege JP Chavaudra J , et al. Experimental assessment of out-of-field dose components in high energy electron beams used in external beam radiotherapy. J Appl Clin Med Phys. 2015;16(6):435-448. 10.1120/jacmp.v16i6.5616 PMC5691002 26699572

[37] Haghparast A Amiri F Yarahmadi M Rezaei M . The peripheral dose outside the applicator in electron beams of an Elekta linear accelerator. Australas Phys Eng Sci Med. 2018;41(3):647-655. 29943310 10.1007/s13246-018-0660-9

[38] Das K Cramb J Millar R , et al. Levels of leakage radiation from electron collimators of a linear accelerator. Med Phys. 1990;17(6):1058-1063. 2126336 10.1118/1.596456

[39] Pennington EC Jani SK Wen BC . Leakage radiation from electron applicators on a medical accelerator. Med Phys. 1988;15(5):763-765. 3141759 10.1118/1.596191

[40] Wen B-C Pennington EC Hussey DH Jani SK . Alopecia associated with unexpected leakage from electron cone. Int J Radiat Oncol Biol Phys. 1989;16(6):1637-1641. 2722601 10.1016/0360-3016(89)90974-7

[41] Edimo P Clermont C Kwato M Vynckier S . Evaluation of a commercial VMC++ Monte Carlo based treatment planning system for electron beams using EGSnrc/BEAMnrc simulations and measurements. Phys Med. 2009;25(3):111-121. 18722148 10.1016/j.ejmp.2008.07.001

[42] O'Shea TP Sawkey DL Foley MJ Faddegon BA . Monte Carlo commissioning of clinical electron beams using large field measurements. Phys Med Biol. 2010;55(14):4083-4105. 20601775 10.1088/0031-9155/55/14/009

[43] Karbaf M Yazdi MHH Ghorbani M Abdollahi S . Assessment of radiation leakage from treatment applicator of Siemens Primus Plus and Siemens Artiste linear accelerators. J Cancer Res Ther. 2019;15(1):216-222. doi:10.4103/jcrt.JCRT_1096_16 30880781

[44] Perec A Kubo H . Radiation leakage through electron applicators on Clinac-1800 accelerators. Med Phys. 1990;17(4):715-719. 2215419 10.1118/1.596472

[45] Schneider AJ . Radiation leakage from electron applicator assembly on a linear accelerator. Med Phys. 1982;9(5):761-762. 7155080 10.1118/1.595125

[46] He H Zhang Y Wang J Chen X Yang Y Wang J . Shielding effect of a lead apron on the peripheral radiation dose outside the applicator of electron beams from an Elekta linear accelerator. J Appl Clin Med Phys. 2021;22(1):327-336. 33296548 10.1002/acm2.13089 PMC7856487

[47] Ebert MA Hoban P . A Monte Carlo investigation of electron-beam applicator scatter. Med Phys. 1995;22(9):1431-1435. 8531868 10.1118/1.597414

[48] Van Battum L Huizenga H . On the initial angular variances of clinical electron beams. Phys Med Biol. 1999;44(11):2803-2820. 10588286 10.1088/0031-9155/44/11/309

[49] Huizenga H Storchi P . The in-air scattering of clinical electron beams as produced by accelerators with scanning beams and diaphragm collimators. Phys Med Biol. 1987;32(8):1011-1029. 3628481 10.1088/0031-9155/32/8/005

